# Mad1 influences interphase nucleoplasm organization and chromatin regulation in *Drosophila*

**DOI:** 10.1098/rsob.180166

**Published:** 2018-10-17

**Authors:** Natacha Raich, Souhir Mahmoudi, Doruk Emre, Roger E. Karess

**Affiliations:** CNRS, Institut Jacques Monod, UMR7592, Université Paris Diderot, Sorbonne Paris Cité, Paris Cedex 13 75205, France

**Keywords:** chromatin, nuclear pore complex, Tpr, Polycomb, spindle assembly checkpoint, mitosis

## Abstract

The *Drosophila* Mad1 spindle checkpoint protein helps organize several nucleoplasmic components, and flies lacking Mad1 present changes in gene expression reflecting altered chromatin conformation. In interphase, checkpoint protein Mad1 is usually described as localizing to the inner nuclear envelope by binding the nucleoporin Tpr, an interaction believed to contribute to proper mitotic regulation. Whether Mad1 has other nuclear interphase functions is unknown. We found in *Drosophila* that Mad1 is present in nuclei of both mitotic and postmitotic tissues. Three proteins implicated in various aspects of chromatin organization co-immunoprecipitated with Mad1 from fly embryos: Mtor/Tpr, the SUMO peptidase Ulp1 and Raf2, a subunit of a Polycomb-like complex. In primary spermatocytes, all four proteins colocalized in a previously undescribed chromatin-associated structure called here a MINT (Mad1-containing IntraNuclear Territory). MINT integrity required all four proteins. In *mad1* mutant spermatocytes, the other proteins were no longer confined to chromatin domains but instead dispersed throughout the nucleoplasm. *mad1* flies also presented phenotypes indicative of excessive chromatin of heterochromatic character during development of somatic tissues. Together these results suggest that *Drosophila* Mad1, by helping organize its interphase protein partners in the nucleoplasm, contributes to proper chromatin regulation.

## 1. Introduction

Mad1 is a key component of the mitotic checkpoint, the mechanism that delays anaphase onset until all chromosomes have properly attached to the spindle. Kinetochore-bound Mad1 acts as a catalyst, helping the protein Mad2 bind to the mitotic regulator Cdc20 and thus generate the anaphase inhibitor [1]. In interphase, Mad1 and Mad2 are usually described as localizing to the nuclear envelope (NE) [2–4], by specifically binding the nucleoporin Tpr, a component of the inner basket of the nuclear pore complex (NPC), [5–8]. This interaction is conserved in yeast, plants and metazoans.

NPC-associated Mad1 may contribute to the proper regulation of mitosis, by pathways that are distinct from Mad1's role at the kinetochore. In the closed mitosis of yeast, Mad1 helps regulate the nuclear import of certain mitotic regulators [9,10]. In human cells, Mad1 protein is stabilized by its association with Tpr, helping to promote kinetochore recruitment of Mad1 and Mad2 [11]. NPC-bound Mad1 is also reported to be a source of anaphase inhibitor prior to the assembly of functional kinetochores [7].

Whether interphase Mad1 has other functions in the interphase nucleus has not been addressed. However, published images of cells from several model organisms suggest that interphase Mad1 may not be restricted to the NE, but may also have a significant nucleoplasmic component [4,7,12–15]. (A pool of extranuclear, Golgi-associated interphase Mad1 has also been described in mammalian cells, with a role in protein secretion [16]).

Here we report that, in *Drosophila*, Mad1 localizes to intranuclear structures in many cell types, including terminally differentiated postmitotic cells. In primary spermatocytes, Mad1 helps assemble a previously undescribed structure that is associated with, but distinct from, the chromatin of these cells. Finally, genetic evidence implicates Mad1 in the establishment or maintenance of proper chromatin conformation and gene expression during *Drosophila* development, apparently independently of its mitotic function.

## 2. Results

### 2.1. Nucleoplasmic Mad1 is widespread in *Drosophila* tissues, and forms a prominent intranuclear structure in spermatocytes

**Figure 1.**
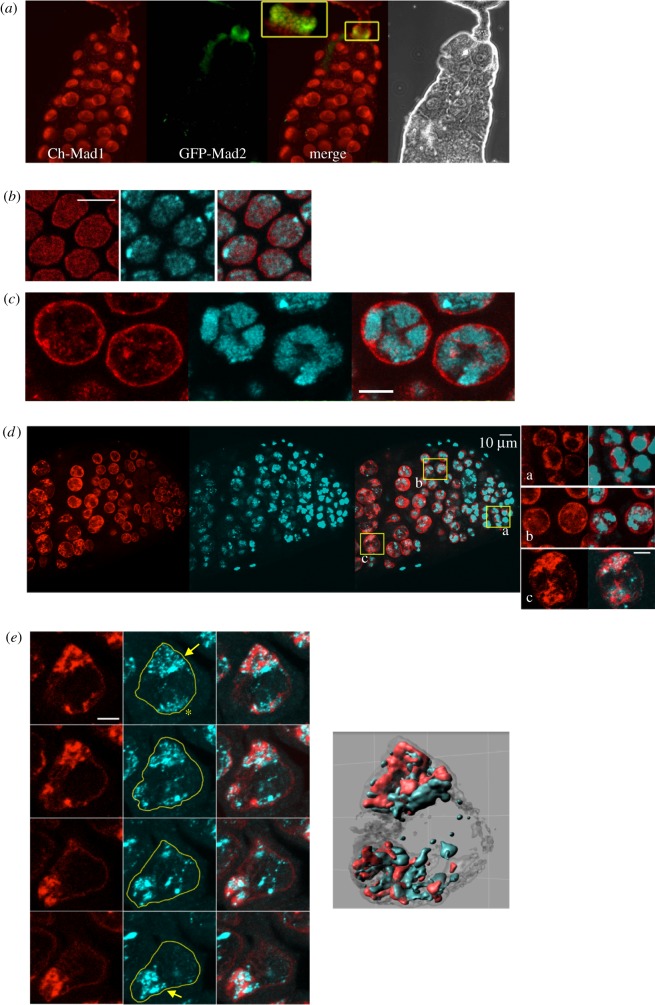
Mad1 is present in the nucleoplasm of both mitotic and postmitotic cells. (*a*) Mad1, but not Mad2, is expressed in all nuclei of larval salivary glands, both mitotic and postmitotic (polytene). Live image of 3rd instar larval salivary gland expressing mCherry-Mad1, and GFP-Mad2. Mad1 labels every nucleus in the tissue, including the postmitotic polytene nuclei, whereas Mad2 is restricted to the anterior ring of mitotically active diploid cells (inset), precursors to the adult salivary gland. See also electronic supplementary material, figure S1. (*b–e*) Mad1 is present both at the nuclear envelope and deeper within the nucleoplasm of different tissues types. From left to right in each series: confocal image optical projections of Mad1 (red), DNA (cyan) and merge. Bars: 5 µm. (*b*) Cellular blastoderm stage embryos. Maximum intensity projection of 2 × 0.5 µm thick stacks. (*c*) Postmitotic nurse cell nuclei of ovarian follicles (stage 5). Note the presence of Mad1 between the chromatin masses. Maximum intensity projection of 2 × 1 µm thick stacks. (*d*) Proximal tip of testis. Intranuclear Mad1 forms an elaborate structure in developing spermatocytes. In early gonial cells (inset a), Mad1 is mostly at the nuclear periphery. In spermatocytes (insets b and c), Mad1 develops into an elaborate structure (the MINT) associated with, but distinct from, the chromatin. Maximum intensity projection of 2 × 1 µm thick stacks. Bar: main panels, 10 µm, insets, 5 µm. (*e*) Four adjacent serial Z-projections (each comprising three adjacent sections of a 12 × 0.5 µm thick stack) of a single stage 5 spermatocyte showing the relationship of the two autosomal chromatin masses (arrows) and the MINTs. The XY chromatin pair (star) has far less Mad1. (*e*, *right*): A 3D rendering of the same spermatocyte nucleus. See also electronic supplementary material, movie S1.

Using fluorescently tagged Mad1 and Mad2 chimeric transgenes under the control of their native promoters [14,17], we found that Mad1 was present in the nuclei of all examined fly tissues, both mitotic and postmitotic, the latter including larval salivary glands (figure 1*a*; electronic supplementary material, figure S1A), larval and adult muscle, and intestinal epithelial cells (not shown), whereas its partner Mad2 was restricted to mitotic cells (figure 1*a*). The apparently universal presence of Mad1, but not Mad2, in fly nuclei suggested an involvement in a non-mitotic function.

**Figure 2.**
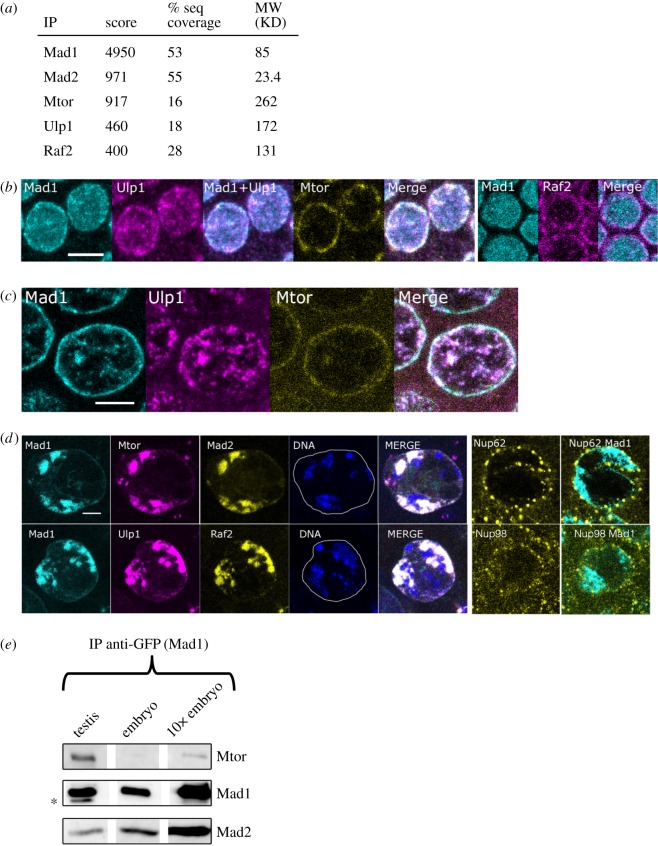
Mad1 coprecipitates with, and can colocalize with Mtor, Ulp1 and Raf2. (*a*) Mass spectrometry analysis of anti-GFP immunoprecipitates of embryos expressing Mad1-GFP in a homozygous *mad1* null mutant background. Score is MASCOT. None of these proteins were detected in immunoprecipitates of embryos expressing free GFP. (*b*) Immunostaining of cellular blastoderm nuclei. Mad1, Ulp1, Mtor and Raf2 all label both the NE and nucleoplasmic particles, but to varying extents. (*c*) Mad1 and Ulp1 colocalize in the nucleoplasm of nurse cell nuclei (same nuclei as in figure 1*c*). (*d*) Ulp1, Raf2, Mtor and Mad2 all substantially colocalize with Mad1 in spermatocytes. Upper panel: Confocal images of a spermatocyte nucleus stained for Mad1, Mtor, Mad2 and DNA. Lower panel: nucleus stained for Mad1, Ulp1, Raf2 and DNA. See also electronic supplementary material, table S1. Right: Nup62 and Nup98 label structures on the NE but do not localize to the MINTs. Maximum intensity projection of 3 × 1 µm thick stacks. Bar: 5 µm. (*e*) Mad1-GFP co-precipitates considerably more Mtor from testis than from embryos. Anti-GFP immunoprecipitates of Mad1-GFP from testis and embryo extracts, analysed for the presence of Mtor and Mad2 by western blotting. The first two lanes were loaded with an amount of immunoprecipitate from testes (corresponding to approx. 100 µg protein extract) and embryos (corresponding to approx. 40 µg protein extract) respectively, that would generate a relatively comparable signal of Mad1-GFP. The rightmost lane, labelled 10× embryo, contains 10-fold more immunoprecipitate material than the middle lane, and yet the coprecipitating Mtor is still weaker than in that of the testis. Thus at least 20-fold more Mtor co-precipitates with a given amount of Mad1-GFP from testis than that from embryos. For comparison, less Mad2 coprecipitates with Mad1 from testes than from embryos. (The band marked by a star is a degradation product.)

Mad1 was found deep within the nucleus as well as at the NE in different tissues at different stages of development (figure 1*b–e*). The relative signal intensity and distribution of Mad1 at the NE versus the nucleoplasm varied considerably depending on the cell type. For example, in cellular blastoderm stage embryos (figure 1*b*, see also figure 2*b*), much of the Mad1 signal was nucleoplasmic, where it presented a granular aspect, distributed both between and upon the DAPI-stained chromatin. In contrast, in postmitotic nurse cell nuclei of adult female egg chambers, Mad1 had a more structured appearance in the nucleoplasm, most evident in the channels between the masses of chromatin (figures 1*c* and 2*c*). In larval neuroblasts, Mad1 was primarily at the NE, but with a diffuse nucleoplasmic component as well [4,14].

A particularly prominent Mad1-containing intranuclear structure was found to assemble within the spermatocytes of adult testis (figure 1*d*). The *Drosophila* testis is a tube filled with developing germ cells and somatic support cells. At the apical end a germ line stem cell divides to generate a spermatogonial cell, which then undergoes four rounds of mitosis, giving rise to a cyst of 16 primary spermatocytes. Over the next several days, during an extended transcriptionally active G2 period, the spermatocytes pass through 6 developmental stages (S1–S6), greatly increasing in size [18] before entering meiosis.

In the mitotic spermatogonial cells, the nuclear volume is largely occupied by chromatin, and Mad1 mainly localized near the nuclear periphery (figure 1*d* inset a). As the early spermatocytes (stages S2–S3) grow, the chromatin separates into three distinct masses (corresponding to the two major autosome bivalents and the XY pair) [18]. In these cells, Mad1 levels noticeably decreased at the NE, and began to form reticular patches associated with the two autosomal chromatin domains (figure 1*d*, insets b, c; electronic supplementary material, figure S1C). Serial optical sections and three-dimensional (3D) reconstruction of stage S4–S5 spermatocytes (figure 1*e*; electronic supplementary material, movie S1) revealed Mad1 to be intimately associated with, but distinct from, the autosomal chromatin masses, with a smaller patch associated with the XY chromatin. We call these structures MINTs (Mad1-containing IntraNuclear Territories).

### 2.2. Mad1 associates with Mtor, Mad2, Ulp1 and Raf2

To better understand Mad1's potential role in interphase nuclei, we looked for proteins that co-immunoprecipitated with Mad1 but were not implicated in the regulation of mitosis by the spindle checkpoint. Specifically, we examined by mass spectroscopy anti-GFP immunoprecipitates from embryos expressing Mad1-GFP in a *mad1* null genetic background [14]. This analysis reproducibly identified four proteins that coprecipitated with Mad1 (figure 2*a*): Mad2 and Megator (Mtor, the fly homolgue of Tpr), known partners of Mad1 in all studied eukaryotes; and two new proteins, the SUMO peptidase Ulp1 (CG12359, homologue of SENP1/2 in mammals) and Raf2 (CG4877), a little-studied protein possessing a MYND zinc finger motif. This protein was initially identified as a subunit of a Polycomb-like complex called RAF [19].

Mtor/Tpr homologues in other eukaryotes have been implicated in many nuclear activities, including nucleocytoplasmic transport, RNP processing, chromatin organization and DNA repair [20–27]. Besides being a component of the NPC basket, where in mammalian cells it is required for maintaining nuclear pore-associated heterochromatin exclusion zones [28], Tpr has been reported to form nuclear structures distinct from the NPC in yeast [25], and deep within the nucleoplasm in mammals and flies [29–32]. In *Drosophila* cell lines, Mtor associates with subsets of chromatin called nucleoporin associated regions (NARs) [24]. It is not known if the various roles attributed to Tpr are the same in these other structures as at the NPC, nor is their physical relationship to the NPC basket understood.

Ulp1 and its orthologues have been localized to the NPCs, and to a lesser extent in the nucleoplasm in fly cells [33], mammalian cells [34,35] and yeast [36], where it has been shown to bind to the Tpr homologue Mlp1/2 [36,37]. Proteins modified by SUMOylation participate in many nuclear processes [38] including regulation of chromatin [39], DNA repair [37], mRNP processing and transport through the NPC [22]. *Drosophila* Ulp1, like most SUMO peptidases, possesses a conserved C-terminal catalytic domain responsible for its desumoylating activity [40,41]. However, *Drosophila* Ulp1 also contains a long N-terminal 900 residue extension with no recognizable homology to known proteins.

### 2.3. Mad1 and its partner proteins colocalize to different extents in different tissues

We examined the localization of these candidate partner proteins relative to Mad1 in blastoderm embryos, nurse cells and spermatocytes (figure 2). In embryos (figure 2*b*), all four proteins were found both at the NE and in the nucleoplasm, but to varying extents. Mtor primarily localized to the NE, as has been reported by others [32,42]. Raf2 also localized principally (though not exclusively) at the NE, whereas Ulp1 was prominent in nucleoplasmic particles whose distribution resembled that of nucleoplasmic Mad1. In nurse cell nuclei (figure 2*c*), Mtor was again far more prominent at the NE, while Ulp1 distribution appeared similar to that of Mad1 in the intranuclear structures, and to a lesser degree at the NE.

By contrast, in spermatocytes, Mad1, Mtor, Ulp1 and Raf2 (as well as Mad2) all substantially colocalized within the chromatin-associated MINTs (figure 2*d*; electronic supplementary material, table S1). In fact, during the development of the spermatocytes, the MINTs, rather than the nuclear periphery, were the principal structures containing these proteins. We considered the possibility that MINTs might correspond to some kind of unusual redeployment of NPCs within spermatocyte nucleoplasm and therefore might contain other nucleoporins besides Mtor/Tpr. We therefore examined four other nucleoporins: Nup62, Nup98 (figure 2*d*), Nup107 and Nup153 (not shown). We were particularly interested in Nup98 which has been shown to bind specific chromatin regions distant from the NE [43]. However, none of these proteins colocalized with the MINTs (figure 2*d*). Thus while MINTs contain components of the NPC basket, they are compositionally distinct from NPCs.

The substantial colocalization of Mad1 and Mtor in spermatocytes correlated with a significant increase (at least 20-fold) in the amount of Mtor that co-immunoprecipitated with a given amount of Mad1-GFP from testis extracts compared to embryo extracts (figure 2*e*). This result, combined with the immunostaining analysis of figure 2, suggests that the different tissue-specific distributions of Mad1 and Mtor may correspond to different degrees of physical interaction between the two proteins, and further suggests that the Mad1–Mtor interaction is developmentally regulated, changing both quantitatively and spatially, as a function of cell type.

### 2.4. Mad1, but not Mad2, is necessary for the assembly of MINTs

**Figure 3.**
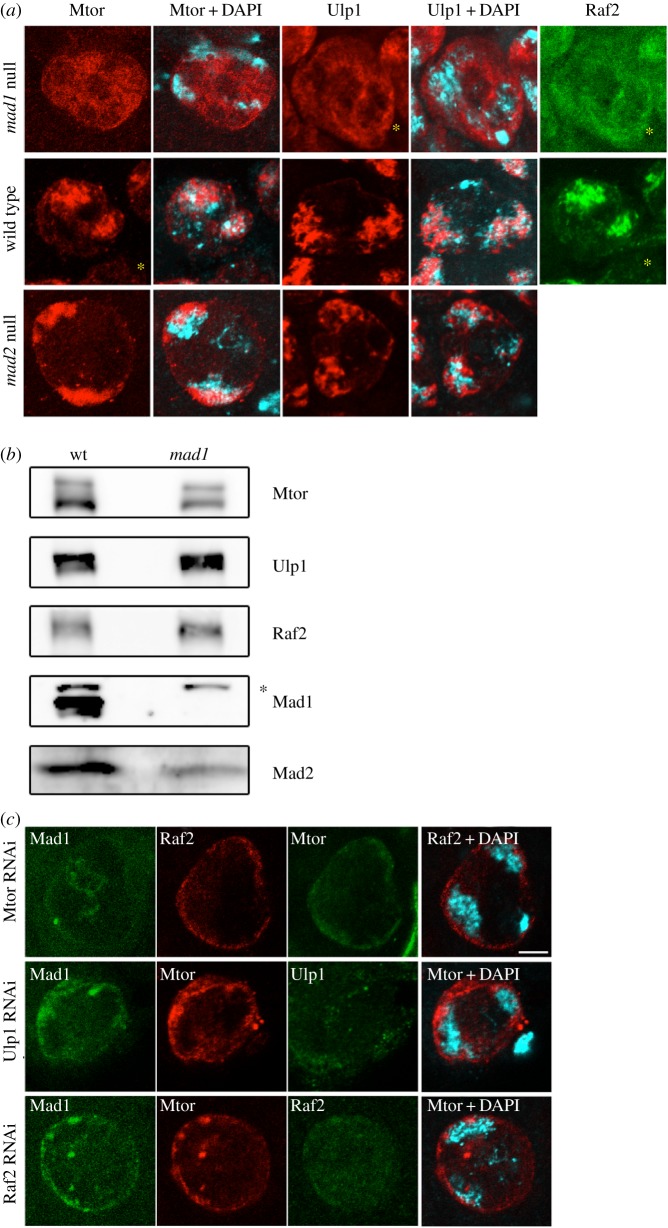
MINTs require Mad1, Mtor, Ulp1 and Raf2. (*a*) MINT components disperse in the absence of Mad1. Stage 5 spermatocyte nuclei from *mad1* (top row), wild-type (middle) or *mad2* (bottom) flies, stained for the different MINT proteins. In *mad1* spermatocytes, Mtor, Ulp1 and Raf2 are distributed unevenly throughout the nucleoplasm. MINTs in *mad2* null spermatocytes are largely intact. Stars indicate doubly labelled cells. (*b*) Western blot of whole tissue extracts from wild-type and *mad1* testis. MINT protein levels are largely maintained in the *mad1* mutant. (The band marked by a star is a background protein detected by anti-Mad1.) See also electronic supplementary material, figure S2. (*c*) Depleting Mtor, Ulp1 or Raf2 eliminates the MINT and reduces signals from the remaining MINT components. Residual proteins associate mostly with the nuclear envelope. Stage 4 or 5 spermatocytes, depleted and stained as indicated. Maximum projections of 4 × 0.5 µm stacks. Bar: 5 µm. See also electronic supplementary material, figure S3.

To assess the structural contribution of Mad1 and Mad2 to the MINTs, we examined the behaviour of the other MINT components in spermatocytes of *mad1* and *mad2* homozygous null mutant flies, both of which are viable in *Drosophila* [14,44]. In *mad1* mutant spermatocytes, MINT-like chromatin-associated structures were no longer detectable. Instead, Mtor, Ulp1 and Raf2 were redistributed diffusely though unevenly throughout the nucleoplasm (figure 3*a*; see also electronic supplementary material, table S1). Western blots of wild-type and *mad1* testis extracts (figure 3*b*; electronic supplementary material, figure S2) revealed no obvious changes in the abundance of these three proteins (though Mad2 levels were slightly reduced). Interestingly, a fraction of Mtor, Ulp1 and Raf2 still localized at the NE in some *mad1* spermatocytes. In fact, their levels at the NE appeared higher in the *mad1* mutants than in wild-type spermatocytes. Thus, the association of these three partner proteins in the MINTs is Mad1-dependent, whereas their localization at the NE is not.

By contrast, the MINTs of *mad2* mutant spermatocytes remained largely intact, compared to those observed in *mad1*: Mtor and Ulp1 still localized in structures associated with the two chromatin masses (figure 3*a*). Although some minor differences with wild-type were detectable, such as more pronounced labelling of the MINT components at the NE, and a tendency for the MINTs themselves to be slightly less robust, we believe this is a secondary effect, as Mad1 protein levels are slightly lower in *mad2* mutants [45]. Thus, Mad1 plays an essential role in organizing Mtor, Ulp1 and Raf2 into a structure specifically associated with the chromatin domains in spermatocytes, whereas its mitotic partner Mad2, despite being recruited to the MINTs, does not.

### 2.5. Depletion of Mtor, Ulp1 or Raf2 disrupts the MINTs in a different manner from the *mad1* mutant

**Figure 4.**
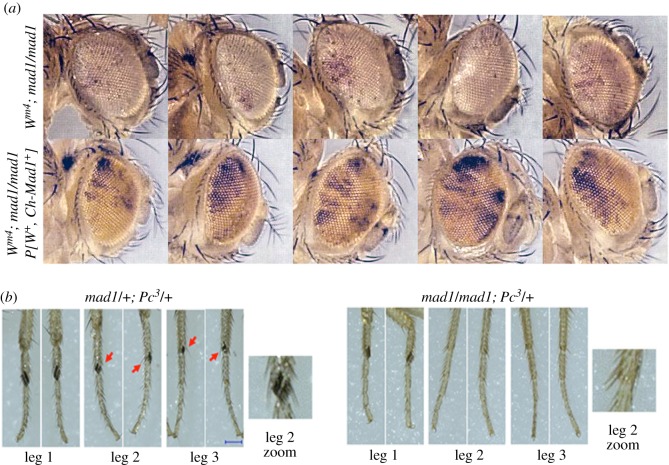
Aspects of chromatin conformation are altered in *mad1* mutants. (*a*) *mad1* enhances heterochromatin-mediated extinction of *white* gene expression caused by the *w^m4^* allele. Examples of eye pigmentation of *w^m4^* flies in homozygous *mad1* mutant (top) and genetically identical siblings additionally expressing Cherry-Mad1 from a transgene and therefore wild-type for *mad1.* The dark pigmentation of eye facets is from the variegating *w^m4^* allele; the uniform pale yellow background of eyes in the second row is derived from a weakly expressing *w^+^* marker carried by the Cherry-Mad1 transgene. The colour contrast of the images has been adjusted to enhance the difference between the background yellow and the darker facets. See Materials and methods for details. See also electronic supplementary material, figure S4. (*b*) *mad1* suppresses the homeotic transformations of Polycomb mutant *Pc^3^*. *Pc^3^* flies with wild-type Mad1 frequently have transformations of L2 and L3 legs into L1, carrying sex combs (arrows). *mad1 Pc^3^* flies display far fewer leg transformations. See also table 1 for statistical analysis.

We also investigated how RNAi depletion of Mtor, Ulp1 and Raf2 proteins would affect Mad1 and the MINTs (figure 4*c*; electronic supplementary material, figure S3), using specific UAS-dsRNA constructs and the *Bam*-Gal4 driver [46] that expresses specifically in the male germ line in late spermatogonia and early spermatocytes. Substantial depletion of Mtor eliminated the MINTs: Mad1, dRaf2, Ulp1 and Mad2 (not shown) no longer localized to chromatin-associated structures. However, unlike in the *mad1* mutant, these proteins did not disperse throughout the nuclear volume. Instead, their overall signals were greatly reduced (figure 3*c*; electronic supplementary material, figure S3), and the remaining signals were largely confined to the NE where they colocalized with the residual Mtor protein (figure 3*c*). Somewhat unexpectedly, depletion of Raf2 or Ulp1 had a similar consequence. Each depleted component profoundly diminished or eliminated the MINT structure, and the remaining proteins, in reduced amounts, colocalized at the nuclear periphery.

**Table 1.** Homozygous *mad1* suppresses the leg transformations of *Polycomb* allele *Pc*^*3*^.

	Polycomb phenotype					
	leg 1		leg 2		leg 3	
genotype	% with comb	average # teeth/comb	% with comb	average # teeth/comb^a^	% with comb	ave # teeth/comb^a^
*mad1*/+; *Pc^3^*	100 (*n* = 14)	11.5	96^b^ (*n* = 23)	5.9	50 (*n* = 24)	2.5
*mad1/mad1*; *Pc^3^*	100 (*n* = 19)	11.3	41^b^ (*n* = 22)	2.0	5 (*n* = 22)	1.0
*mad2/mad2*; *Pc^3^*	n.d.		88^c^ (*n* = 14)	6.3	79 (*n* = 14)	2.7

^a^On legs with combs.

^b^Total ectopic teeth: *mad1/mad1* versus *mad1/+*; Student's one-tailed *t*-test *p* < 10^−4^.

^c^*mad2/mad2* versus *mad1/+*; one-tailed *t*-test: *p* = 0.2.

Significance calculated for number of transformed legs or for total number of ectopic teeth.

In summary, of the five MINT components (Mad1, Mad2, Mtor, Ulp1 and Raf2) identified here, all but Mad2 are necessary, but not sufficient, for assembly or maintenance of a morphologically recognizable MINT in the spermatocyte nucleus. However, the removal of Mad1 causes the other MINT components to redistribute within the nucleoplasm, whereas the depletion of Mtor, Ulp1 or Raf2 eliminates the MINTs and leads to an overall reduction in nuclear levels of the other MINT proteins (which might suggest an effect on their retention or stability within the nucleus).

### 2.6. *Mad1* behaves genetically as a modifier of chromatin conformation

Mtor/Tpr, Ulp1 and Raf2 have all been previously implicated in aspects of chromatin regulation. The ubiquitous presence of Mad1 in the interphase nucleoplasm of fly cells, and its capacity (in spermatocytes) to influence the intranuclear localization of Mtor/Tpr, Ulp1 and Raf2, suggested that Mad1 might contribute to proper chromatin conformation, an activity quite different from its role in the spindle assembly checkpoint.

To test for possible influence of Mad1 on chromatin conformation more generally in fly tissues, we employed two sensitive genetic assays in which visible cuticular phenotypes in the adult fly reveal structural alterations in epigenetic chromatin packaging.

In the first test, we asked if *mad1* was a modifier of heterochromatic position effect variegation (PEV) of the *w^m4^* allele of the *white* locus, which regulates pigmentation in the adult eye. In *w^m4^* flies, the normally euchromatic *white* gene is juxtaposed near centric heterochromatin. Clonal patches of pigmented and unpigmented eye tissue reflect, respectively, the euchromatic and heterochromatic status of the *w^m4^* locus (reviewed in [47]). When *w^m4^* was placed in a homozygous *mad1* null mutant background, the typical eye pigment levels were reduced (figure 4*a*; electronic supplementary material, figure S4), relative to that of *mad1/+* heterozygotes or of *mad1/ mad1* homozygotes complemented by expression of a wild-type *Mad1* transgene. That is, *mad1* was acting as a recessive enhancer of variegation, promoting excessive heterochromatinization around *w^m4^*.

In the second assay, we asked if *mad1* could influence Polycomb-mediated gene repression *in vivo* [48]. Flies heterozygous for the *Pc^3^* allele [49] have a reduced capacity to silence some chromatin, and famously display frequent homeotic transformations of second and third leg pairs into ‘first legs’ as revealed by the presence of ectopic sex combs (normally found only on first legs). In *Pc^3^* flies carrying one wild-type allele of Mad1, 96% (22/23) of scored second legs (L2s) and 50% (12/24) of third legs (L3s) had ectopic sex combs, indicating their partial transformation into first legs (L1s). By contrast, *Pc^3^* flies that were homozygous for *mad1* showed significantly reduced frequency of L2–L1 (41%) and L3–L1 (5%) leg transformations (figure 4*b* and table 1). Moreover, the sex combs on those transformed L2s were smaller, averaging only 1–2 teeth/comb instead of 5–6 teeth/comb in the *mad1/+* heterozygotes (table 1). Importantly, no such suppression of the *Pc^3^* phenotype was seen in *mad2* null homozygote flies, strongly arguing that the effect of *mad1* is unrelated to its mitotic function.

In summary, both genetic assays indicated an organism-wide tendency for chromatin to adopt a more heterochromatic or silent conformation in the absence of Mad1. These results suggest that wild-type Mad1 (in conjunction with its interphase partners) normally helps establish or maintain an *active* chromatin state in many (and perhaps all) cell types of the fly.

## 3. Discussion

This study presents evidence that: (i) Mad1 is present in the nucleoplasm of both mitotic and postmitotic cells in *Drosophila*; (ii) Mad1 associates with at least three proteins, Mtor/Tpr, Ulp1 and Raf2, with known or suspected roles in chromatin packaging; (iii) Mad1 helps organize a prominent nucleoplasmic structure (the MINTs) in spermatocytes containing these three proteins; and (iv) Mad1 can influence chromatin conformation, and thus gene expression, in the imaginal eye and leg tissues during development.

### 3.1. Mad1 helps assemble Mtor, Ulp1 and Raf2 into a nucleoplasmic structure distinct from the NPC in spermatocytes

MINTs do not seem to have been described previously in the literature. Various transcription factors and accessory proteins localize to the spermatocyte chromatin masses, for example [50–53], but none resembles the structures labelled with Mad1 and the other MINT components described here, which enlace the autosomal chromatin masses.

A striking feature of the spermatocyte MINT is how its integrity depends on Mad1. In its absence, Mtor disperses throughout the nucleoplasm. In cultured mammalian cells, yeast and plants, Tpr has been shown to anchor Mad1 to the NE, via the NPC basket [5,7–9]. Yet unlike the NPC basket, in the MINTs it is Mad1 that appears to assemble Mtor into a MINT. A model to explain the developmentally regulated, Mad1-dependent redeployment of NE-associated Mtor into MINTs as the gonial cells mature into spermatocytes would be to posit that the two proteins physically interact in a regulated manner. This interaction would be distinct from the ‘constitutive’ binding of Mad1 and Mtor responsible for Mad1's presence at the NE, and would also allow Mad1 and Mtor to generate higher-order crosslinked structures, corresponding to the MINTs. One prediction of the model is that the organization of these proteins within the MINTs will not be the same as that found at the NE. This, in turn, suggests that MINTs may perform a specialized function distinct from that carried out by Mtor and its partners at the NPC basket.

The proximity of MINTs to the autosomal chromatin masses suggests a physical link with a chromatin-associated protein. Removing Mad1 appears to rupture this link, but Mad1 itself is unlikely to be binding directly to chromatin. Intriguingly, Mtor, Ulp1 and Raf2 were all found to partially copurify with a novel Polycomb-like complex called RAF [19]. Mad1 was not reported in this complex (which was isolated from cultured *Drosophila* cells), but one might imagine that Mad1 in spermatocytes is required to stabilize the association of Mtor, Ulp1 and Raf2 with chromatin-bound RAF, and thus indirectly provides the link between the MINTs and chromatin.

### 3.2. How might Mad1 help maintain proper chromatin conformation?

All three non-checkpoint proteins associating with Mad1 (both by co-immunoprecipitation from embryo extracts and by colocalization in spermatocyte MINTs) have been implicated in the regulation of chromatin or chromatin-associated factors: Mtor was found to preferentially associate with chromatin domains enriched for markers of active transcription [24]; Ulp1 activity reverses SUMOylation, a post-translational modification regulating the activities of many nuclear proteins, including some Polycomb complexes [54,55]; and Raf2 is a core component of the RAF Polycomb-like complex [19]. In addition, Tpr and Ulp1 are major players in other nuclear functions, such as mRNP assembly and nucleocytoplasmic transport [38], perturbation of which may indirectly affect chromatin conformation as well.

Both of the genetic tests we employed to assay a possible role for Mad1 on chromatin conformation in somatic cells during development revealed a tendency for *mad1* mutants to have excess chromatin of a heterochromatic character. The suppression by *mad1* of the extra sex comb phenotype of *Pc^3^* suggests that in these flies genes normally subjected to Polycomb repression were still repressed even when Pc activity was reduced by the *Pc^3^* allele. Similarly, the enhanced variegation of *w^m4^* in *mad1* mutant flies indicates a trend towards heterochromatinization of the *white* gene on the X chromosome. Indeed nearly all genetically defined modifiers of PEV have proven to be modifiers of chromatin [47]. Thus the wild-type activity of Mad1 seems to contribute to the establishment or maintenance of proper ‘open’ chromatin conformation. The fact that a *mad2* null mutation did not alter the Polycomb phenotype supports our conclusion that this new Mad1 activity is unrelated to its role in the spindle assembly checkpoint.

Although nucleoplasmic Mad1 is present in all fly tissues examined, only in spermatocytes does Mad1 form such prominent structures. The nuclear organization of spermatocytes is atypical. They have some of largest diploid nuclei in the fly life cycle, and their chromatin territories are well-separated in the nuclear volume, which may be why the MINTs are detectable as discrete structures in these cells. In addition, only in spermatocytes does the majority of Mtor/Tpr colocalize with nucleoplasmic Mad1, rather than at the NE.

On the other hand, at least some of the much smaller nucleoplasmic speckles of Mad1 seen, for example, in early embryonic nuclei (figure 1) appear to colocalize with subsets of Ulp1, Raf2 and Mtor signals (the latter particularly at the NE), consistent with the proteomic analysis of figure 2*a*. Thus in these nuclei any structures containing all four components would seem to involve only a small minority of these proteins. There might also exist an assembly in which Mtor is absent (or present but at reduced stoichiometry), as suggested by the co-immunoprecipitation analysis of figure 3. In spermatocytes, the removal of Mad1 releases Mtor, Ulp1 and Raf2 from their normal proximity to the chromatin, and these proteins redistribute throughout the nucleoplasm. But changes in the nuclear distribution of Mtor, Ulp1 or Raf2 signals caused by depletion of Mad1 in diploid cell types other than spermatocytes would affect only a fraction of the corresponding fluorescent signals and therefore would escape detection by conventional microscopy.

Accordingly, we suggest therefore that smaller MINT-like complexes, dependent on Mad1, do exist in all cell types of the fly, carrying out specific functions that help establish or maintain proper chromatin states during development. One can imagine two (non-exclusive) general sets of consequences for chromatin in *mad1* mutants: (i) The chromatin sites normally under the localized regulatory influence of Mtor, Ulp1 and Raf2 would find that regulation reduced; (ii) chromatin elsewhere in the nucleus normally not associated with these proteins would become exposed to their activities. Determining which scenario is predominantly responsible for the excess heterochromatic character of chromatin in *mad1* mutants may not be straightforward, because chromatin associated with Mtor reportedly tends to be ‘open’ [24] while chromatin influenced by Polycomb group complexes (which one might expect to be modified by the relocation of Ulp1 and Raf2) tends to be of heterochromatic character. In this regard, we note that genetic depletion of Kdm2, another core subunit of RAF, enhances the Polycomb phenotype of *Pc^3^* [19], whereas depletion of Mad1, as we have shown, suppresses it.

## 4. Material and methods

### 4.1. *Drosophila* stocks

The *mad1^1^* null allele, and Mad1-GFP and -Cherry transgenes are described in Emre *et al.* [14]. ‘Wild-type’ flies in the described experiments are either *mad1^1^/*CyO heterozygotes, or genotype *P*[*Mad1-GFP*]; *mad1^1^/mad1^1^*, where the tagged transgene, inserted either on chromosome X or chromosome 3, is the wild-type allele. The *mad2^p^* null allele is from Buffin *et al.* [44], GFP-Mad2 transgene from Buffin *et al.* [17], and *ptc-GAL4 UAS-GFP* was a gift from A. Guichet (IJM). The position-effect-variegation (PEV) allele *white-mottled4* (*w^m4^*) was a gift from S. Ronsseray and C. Carré (Univ. PM Curie, UMR7622, Paris). The *Pc^3^* Polycomb allele is from the Bloomington Stock Center. The dsRNA stocks are from Vienna *Drosophila* RNAi Center, VDRC : *Mtor*^RNAi-1^ (ID 110218), *Mtor*^RNAi-2^ (ID 24265), *Ulp1*^RNAi-1^ (ID 106625), *Ulp1*^RNAi-2^ (ID 31744), *Raf* ^RNAi-1^ (ID21966).

### 4.2. RNAi depletion

The Bam-Gal4VP16 [46,56] was used to drive Mtor, Ulp1 or Raf2 UAS-RNAi expression in spermatocytes. RNAi knockdown experiments were carried out by crossing females carrying the desired dsRNA hairpin constructs (on either chromosome 2 or 3) under UAS promotor control to males of genotype *y w; UAS-Gal4/CyO; Bam-Gal4, Mad1-GFP/TM6*. The progeny were raised at 25°C for 2 days then transferred to 29°C to induce expression until birth. (In preliminary studies, the efficiency of depletion of these three proteins, judged by immunofluorescence, was insufficient at 25°C). Testes of young males (less than 24 h post-eclosion) lacking the CyO and TM6 balancers (and therefore carrying the necessary drivers and expression transgenes) were dissected for analysis. Controls were identically treated males lacking the UAS dsRNA transgenes. For both *Mtor*^RNAi-1^ (ID 110218 and ID 24265) and *Ulp1*^RNAi-1^ (ID 106625 and ID 31744), similar results were observed with both lines and this ensures that any observed phenotypes are not due to an off-target effect. Each RNAi experiment was repeated at least three times. The results presented here are from the line *Mtor*^RNAi-1^ (ID 110218) and *Ulp1*^RNAi-1^ (ID 106625). Only one line was available for *Raf2*^RNAi-1^ (ID21966).

### 4.3. Histochemistry and imaging

Testes were dissected in Ringer buffer [18] and fixed in 4% paraformaldehyde in phosphate-buffered saline (PBS) with NP40 0.5% plus two volumes of heptane at room temperature for 30 min [57]. After washes in PBS, testes were next permeabilized in 0.3% Triton X-100 for 30 min and blocked in the same buffer plus 5% NGS (normal goat serum) for 1 h. Testes were incubated in primary antibody overnight at room temperature. All the immunostainings were repeated at least five times on different samples.

In all experiments described here, Mad1 was stained with antibodies to GFP (to detect Mad1-GFP). We have previously confirmed that GFP signal corresponds to endogenous anti-Mad1 labelling [4,14] in fly neuroblasts and early embryos.

The following antibodies and dilutions were used: GFP booster, 1 : 200 (Chromotek); a mixture of monoclonal anti-GPF mouse clones 7.1 and 13.1, 1 : 200 (Roche); rabbit polyclonal anti-GFP (Invitrogen); rat anti-Mtor, 1 : 1000 [24] (gift from A. Akhtar, EMBL); mouse anti-Mtor, 1 : 40 [32] (gift from J. Johansen, Iowa State University); rabbit anti-Raf2, 1 : 200 [19] and guinea pig anti-Ulp1, 1 : 200 [19] (gifts from C. P. Verrijzer, Erasmus University, Rotterdam); rabbit anti-Mad2, 1 : 100 (gift from David Sharp, AECM, New York); mouse anti-NUP153, 1 : 75 (QE5, Abcam); and mouse anti-lamin, 1 : 100 (Developmental Studies Hybridoma Bank). (Note: GFP booster shows a weak cross-reactivity with the Y-loops, ribonucleoprotein structures prominent in spermatocytes of stages 4–5. See electronic supplementary material, figure S1D). Rat anti-Nup62 (1 : 200) [58] was a gift from H. Ohkura (University of Edinburgh, UK) and anti-Nup98 (1 : 100) was from Abcam (2H10). Secondary antibodies were from the Dye-Light conjugated series (1 : 500; Thermo Fisher Scientific). DNA was counterstained with DAPI (1 µg ml^−1^) before coverslips were mounted using CitiFluor AF1 on glass slides. Confocal images were acquired on a Zeiss LSM710 confocal microscope (63×, Plan Apochromatic oil DIC objective lens) using the Zen software (Carl Zeiss). Wild-type and mutant (or RNAi) tissues were always fixed and stained in parallel, mounted on the same slide, and images acquired with the same parameters. The wildfield images of figure 1*a* and electronic supplementary material, figure S1A–C were obtained on a Zeiss AxioImager Z1 microscope.

For visualization of colocalization of confocal images, different image channels were overlaid in the same *Z*-plane. Consequently, a green and red overlay gave rise to yellow hotspots where the two molecules of interest were present in the same pixel locations. Colocalization was quantitated with an intensity correlation coefficient-based method using the Coloc-2 ImageJ plugin. The nucleus was defined using the ROI tool. Pearson's correlation coefficients (PCCs) were collected using the ROI manager to quantify the degree of colocalization between fluorophores [59]. PCC can range from + 1 (denoting perfect positive correlation) to −1 (perfect negative correlation), with 0 indicating no correlation (see electronic supplementary material, table S1).

Images were analysed and processed (contrast adjustments, z-projections) in ImageJ, and Photoshop CS4 (Adobe), and represent maximum intensity projections of z stacks as indicated in the figures. The 3D reconstruction of the spermatocyte nucleus in figure 2*e* and electronic supplementary material, movie S1 was achieved using IMARIS software (Bitplane). The nuclear surface was defined by low level expression of Mad1 at the NE.

### 4.4. Immunoprecipitation and proteomic analysis

All immunoprecipitation (IP) experiments were performed on protein extracts as described [60]. One- to three-hour-old embryos expressing the Mad1-GFP transgenes in homozygous *mad1^1^* null background were harvested, washed and devitellinized. Embryos expressing free GFP from a fly stock of genotype *ptc-GAL4 UAS-GFP* were used as a control. The embryos were lysed in homogenization buffer (10 mM Tris–HCl at pH 7.5, 0.15 M NaCl, 1 mM EDTA, 0.5% NP40), complete ULTRA and phoStop inhibitors (Roche Diagnostics) using a Dounce homogenizer, and the lysates were precleared by centrifugation (2 × 10 min at 20 000×*g*). For co-immunoprecipitation studies and for mass spectrometry analysis, 2 mg of precleared protein extract was immunoprecipitated with 50 µl of µMACS anti-GFP MicroBeads (Miltenyi Biotec, Bergisch Gladbach, Germany) for 30 min at 4°C. The mixture was applied onto a µColumn (Miltenyi) and allowed to run through by gravity flow as described by the supplier. The immobilized beads were washed 4× with 200 µl of homogenization buffer without NP40 and 2× with 200 µl of 20 mM Tris–HCl at pH 7.5 buffer. Proteins were eluted from the beads with 50 µl of 0.5 M NH_4_OH. This procedure routinely immunoprecipitated 70–80% of the total GFP-tagged proteins present in the lysate. For immunoprecipitation of testis extracts, 50 dissected testes from 0–1-day old adults were processed with same conditions as above.

The isolated immune complexes were digested overnight at 37°C with sequencing grade trypsin (12.5 µg ml^−1^, Promega) in 20 µl of 25 mM NH_4_HCO_3_. Digests were analysed by a LTQ Velos Orbitrap coupled to an Easy nano-LC Proxeon system (Thermo Fisher Scientific, Illkirch, France) at the IJM proteomics facility. Data were processed with Proteome Discoverer 1.4 software (Thermo Fisher) coupled to an in-house MASCOT search server (v. 2.3.02, Matrix Science, Boston, MA). False discovery rates for peptide identification were estimated by the Percolator algorithm (Matrix Science). A threshold of 0.01 was used to consider a peptide as identified. Proteomic analysis of immunoprecipitates was performed three times on independent embryonic extracts.

### 4.5. Immunoblotting

Testes were lysed in homogenization buffer (10 mM Tris–HCl at pH 7.5, 0.15 M NaCl, 1 mM EDTA, 1% NP40), complete ULTRA and phoStop inhibitors (both from Roche Diagnostics) and benzonase (Sigma) using a Dounce homogenizer, and the lysates were precleared by centrifugation (2 × 10 min at 20 000×*g*). Total extracts or immunoprecipitates were subjected to western blot (WB) analysis. The following antibodies were used for WB: mouse anti-Mad1, 1 : 500; rat anti-Mtor, 1 : 1000 [24]; rabbit anti-Raf2, 1 : 1000; guinea pig anti-Ulp1, 1 : 1000; rabbit anti-Mad2, 1 : 500. All immunoblot experiments were carried out at least two times on two independent samples.

### 4.6. Genetic tests

To assess the effects of homozygous *mad1* null mutation on PEV of *w^m4^*, four independent lines of *w^m4^*; *mad1^1^/CyO* were generated, using two different stock sources of *w^m4^*, and backcrossed for several generations to an isogenic stock of *y w*; *mad1^1^/CyO*, to minimize the presence of modifiers of PEV from extraneous sources. Individual *w^m4^*/*Y*; *mad1^1^/CyO* males were then crossed to *y w; mad1^1^/+* females, and the eye variegation of sibling daughters of genotype *w^m4^/+; mad1^1^/+* and *w^m4^/+; mad1^1^/ mad1^1^* were compared (homozygous *mad1* mutants are identifiable by the slight roughness (misalignments of the ommatidia) in the eye. In a second set of crosses (used for figure 4*a*), the *w^m4^*/*Y*; *mad1^1^/CyO; +/+* males were crossed to females of a stock of *y w; mad1^1^/mad1^1^*; *P*[*w^+^, Ch-Mad1*]*/+.* Non-Cy female offspring from this cross were either *w^m4^/+; mad1^1^/ mad1^1^*; +/+ (*mad1* mutant) or *w^m4^/+; mad1^1^/mad1^1^*; *P*[*w^+^, Ch-Mad1*]*/+* (wildtype with respect to *mad1*), but otherwise genetically identical. The *P*[*w^+^, Ch-Mad1*] transgene used here carries a weakly expressing *w^+^* gene, conferring a uniform pale orange-yellow colour to the eyes. This coloration is easily distinguishable from the darker mottled facets from *w^m4^* expression. The images presented in figure 4*a* are all siblings from a single such cross. The colour contrast of the images here has been adjusted to enhance the difference between the background orange and the darker facets.

To assess the effects of homozygous *mad1* mutation on the dominant Polycomb-3 (*Pc^3^*) phenotype a stock of *y w*; *mad1^1^/CyO*; *Pc^3^*/*MKRS* was generated, and crossed to *P*[*Mad1-GFP*]; *mad1^1^/mad1^1^*; +/+ flies. Homozygous *mad1* or heterozygous (with one copy of the wild-type *Mad1-GFP* transgene) males carrying *Pc^3^* (non-MKRS) were scored for leg transformations. The number of teeth per extra sex comb were also scored. To test the effects of *mad2* null on *Pc3* phenotype, a stock of *y w*; *mad2^p^ Pc^3^*/*TM6* was generated, and crossed to *y w mad2^p^/TM6.* Leg transformations on *mad2^p^ Pc^3^/mad2^p^* males were scored. Significance was established by one-tailed *t*-test.

## Supplementary Material

Supplemental Figures

## Supplementary Material

Supplemental legends and tables

## Data accessibility

This article has no additional data.
