# Supplementary figures and images for: Mad1 influences interphase nucleoplasm organization and chromatin regulation in Drosophila
Source: Open Biol. 2018 Oct 17;8(10):180166. doi: 10.1098/rsob.180166 (PMC6223205; doi:10.1098/rsob.180166)

Supplemental Fig S1

A

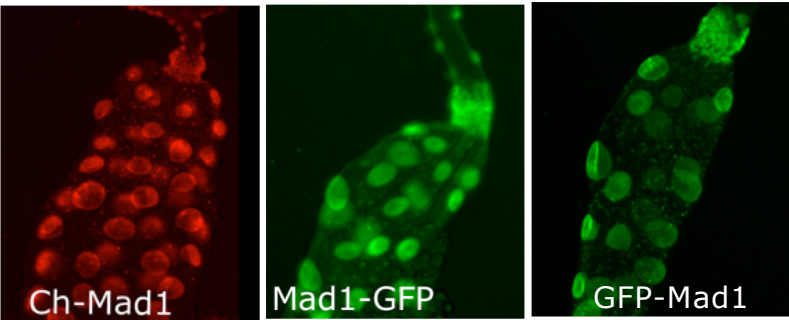

B

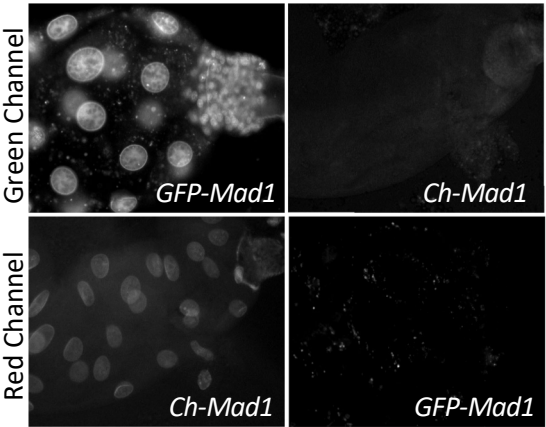

C

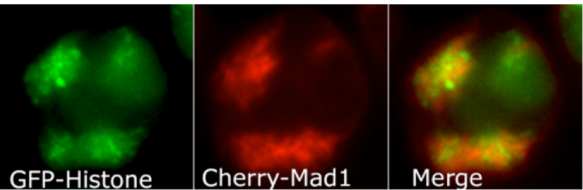

D

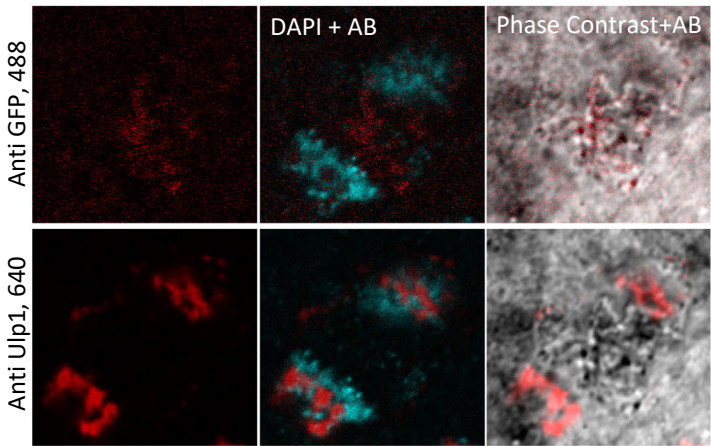

Supplemental Fig S2

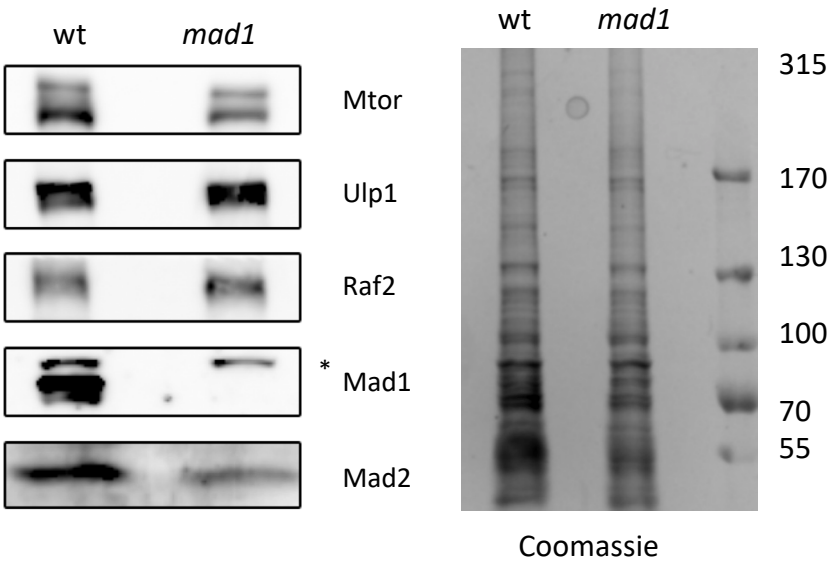

Supplemental Fig S3

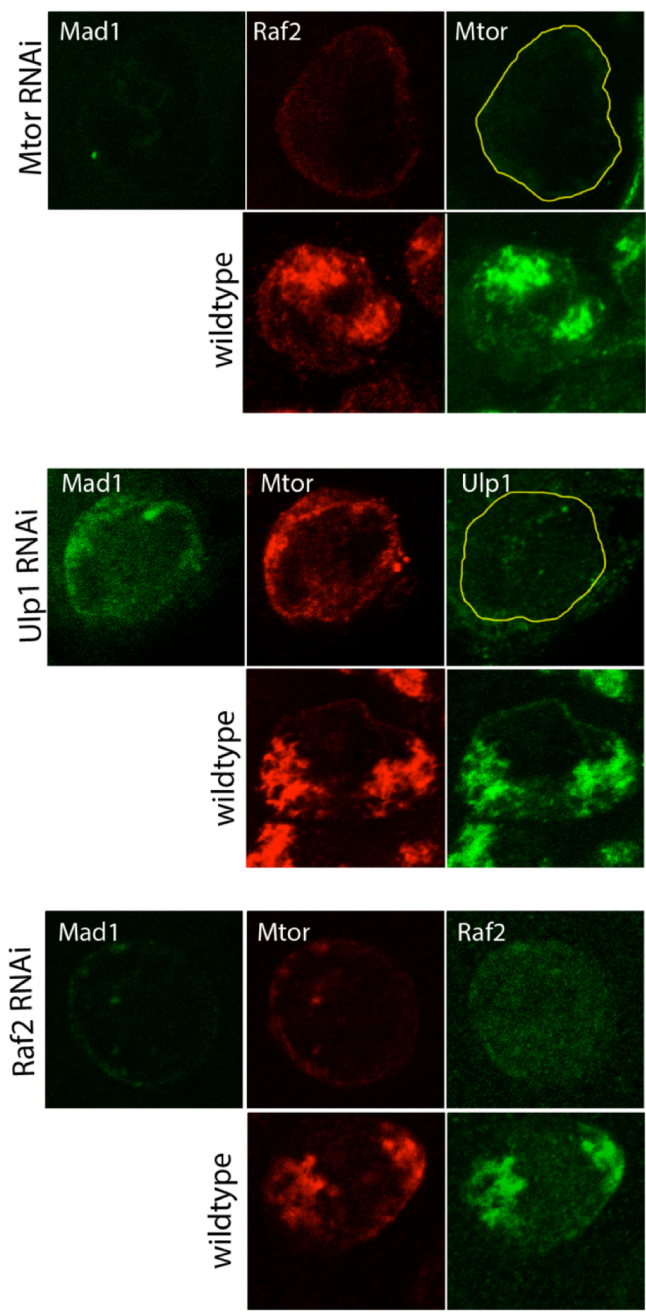

# Supplemental Fig S4

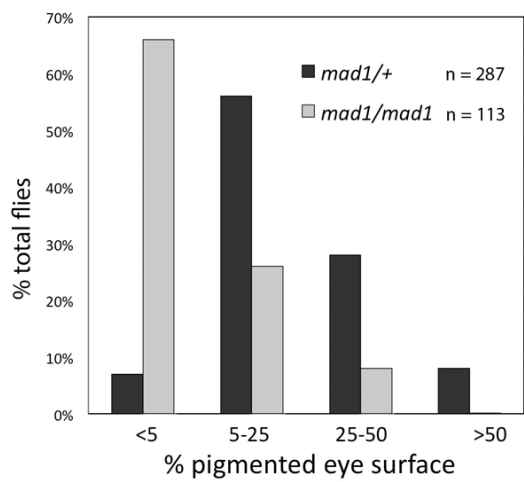

Supplement: Supplemental Figures [file rsob180166supp1.pdf]
