# Supplementary material for: Mad1 influences interphase nucleoplasm organization and chromatin regulation in Drosophila
Source: Open Biol. 2018 Oct 17;8(10):180166. doi: 10.1098/rsob.180166 (PMC6223205; doi:10.1098/rsob.180166)
Supplement: Supplemental legends and tables [file rsob180166supp2.docx]

**Supplemental Movie 1**

Imaris 3-D rendering of the spermatocyte in Fig 2E. DNA is in purple, Mad1 in green.

**Figure S1, accompanying Fig 1.**

1. Postmitotic nuclei of larval salivary glands contain Mad1 regardless of the transgene employed. Cherry-Mad1 (from Fig 1) compared with Mad1-GFP, and GFP-Mad1, independently generated transgenes inserted at different loci. The GFP-Mad1 transgenic construct starts 11 kb upstream of the Mad1 transcription initiation site (and includes the entire adjacent upstream transcription unit), and extends 2 kb downstream of the Mad1 transcription termination site. It was inserted at a *phiC31* site-specific recombination site. The other transgenic constructs start 1.5 kb upstream of Mad1, and were inserted by P-mediated transformation. (See [14] for details).
2. GFP- and Cherry-tagged Mad1 signals in salivary gland nuclei are not artefacts of autofluorescence. The panels show two larval salivary glands, one expressing GFP-Mad1 (top left, bottom right), the other expressing Cherry-Mad1 (top right, bottom left). Identical acquisition conditions were employed, changing only the fluorescent filter. Identical pixel intensity ranges are displayed. Without the appropriate combination of tagged transgene and fluorescent filter, no nuclear signal is detectable.
3. MINTs are not an artefact of fixation. Live image of a single stage 4 or 5 spermatocyte nucleus expressing Ch-Mad1 and GFP-Histone. Compare with fixed images of Mad1-GFP and DAPI staining in Figs 1 and 2.
4. Anti-GFP antibodies do not detect MINT structures in spermatocytes not expressing GFP-tagged Mad1. A single stage-5 spermatocyte from a non-transgenic testis is shown here stained with GFP-Booster (anti-GFP, 488nm), anti-Ulp1 (+ anti-guinea pig, 640nm), and DAPI. The MINT structures are revealed by the anti-Ulp1 but not by the anti-GFP. A very weakly staining structure cross-reacting with the anti-GFP probably corresponds to the Y-loops, visible by phase contrast (see [18]).

**Figure S2, accompanying Fig 3B.**

Whole tissue extracts from wild type and *mad1* testis. *Left*: Western blot from Fig 3. *Right*: The corresponding Coomassie stained gel, indicating comparable protein loading of wild type and *mad1* extracts. MINT protein levels are largely maintained in the *mad1* mutant.

**Figure S3, accompanying Fig 3C.**

"Semiquantitative" images of RNAi depletion of Mtor, Ulp1, Raf2, compared to the corresponding wild type stainings, acquired and processed with identical parameters. In Fig 3C, the signals of the partner proteins were enhanced to reveal their localization. In Fig S3 their levels have not been altered. Contours of the nuclei are indicated for the depletion of Mtor and Ulp1, which otherwise are undetectable.

**Figure S4, accompanying Fig 4A.**

Homozygous *mad1* mutants enhance heterochromatin-mediated extinction of *white* gene expression caused by the *w ^m4^* allele. Quantitation of the extent of *w^m4^* eye pigmentation in wild-type (*mad1*/+) and homozygous *mad1* mutants, “binned” into the indicated groups. The distributions were judged significantly different by Mann-Whitney Rank Sum Test (p< 0.02)

**Table S1**

| **Proteins stained** | **Correlation pairs** | **Pearson’s Correlation Coefficient (PCC)** | |
| --- | --- | --- | --- |
|  |  | **wild type** | ***mad1*** |
| Mad1  Ulp1  Raf2 | Mad1-Ulp1  Mad1-Raf2  Ulp1-Raf2 | 0.73  0.70  0.68 | 0.34 |
| Mad1  Mtor  Mad2 | Mad1-Mtor  Mad1-Mad2  Mtor-Mad2 | 0.63  0.57  0.68 | 0.13 |
| Mad1  Mtor  Ulp1 | Mad1-Mtor  Mad1-Ulp  Mtor-Ulp1 | 0.58  0.57  0.63 | 0.28 |

In three separate immunostainings, wild type or *mad1* mutant spermatocytes were labeled for 3 of the 4 MINT components (first column). The PCC (third and fourth columns) was determined for each of the indicated signal pairs (second column), as described in the Methods. The pairwise signals were highly correlated in the wild type (PCC approx. 0.6-0.7), but low in the *mad1* mutant.
